# Genome surveillance of SARS-CoV-2 variants and their role in pathogenesis focusing on second wave of COVID-19 in India

**DOI:** 10.1038/s41598-023-30815-5

**Published:** 2023-03-22

**Authors:** Poulomi Sarkar, Sarthak Banerjee, Sarbar Ali Saha, Pralay Mitra, Siddik Sarkar

**Affiliations:** 1 CSIR-Indian Institute of Chemical Biology (IICB), Calcutta, WB 700032 India; 2 IICB-Translational Research Unit of Excellence, Cancer Biology and Inflammatory Disorder, Salt Lake, WB 700091 India; 3 Indian Institute of Technology (IIT) Kharagpur, Computer Science and Engineering, Kharagpur, WB 721302 India

**Keywords:** Inflammation, Data mining

## Abstract

India had witnessed unprecedented surge in SARS-CoV-2 infections and its dire consequences during the second wave of COVID-19, but the detailed report of the epidemiological based spatiotemporal incidences of the disease is missing. In the manuscript, we have applied various statistical approaches (correlation, hierarchical clustering) to decipher the pattern of pathogenesis of the circulating VoCs responsible for surge in the incidences. B.1.617.1 (Kappa) was the predominant VoC during the early phase of the second wave, whereas, Delta (B.1.617.2) or Delta-like (AY.x) VoC constitutes majority ($>90.17$%) of the cases during the peak of the second wave. The correlation plot of Delta/Delta-like lineage demonstrates inverse correlation with other lineages including B.1.617.1, B.1.1.7, B.1, B.1.36.29 and B.1.36. The spatiotemporal analysis shows that most of the Indian states were affected during the peak of the second wave due to the Delta surge, and fall under the same cluster. The second cluster populated mostly by north-eastern states and the islands of India were minimally affected. The presence of signature mutations (T478K, D950N, E156G) along with L452K, D614G and P681R within the spike protein of Delta or Delta-like might cause elevation in the host cell attachment, increased transmission and altered antigenicity which in due course of time has replaced the other circulating variants.The timely assessment of new VoCs including Delta-like will provide a rationale for updating the diagnostic, vaccine development by medical industries and decision making by various agencies including government, educational institutions, and corporate industries.

## Introduction

COVID-19 is the calamitous ongoing public health emergency since the 1918 influenza pandemic^1^ which has claimed over 5.4 million of lives while infected more than 279 million population globally as of December 2021 (https://covid19.who.int/table) (Supplementary Table S1). The first COVID-19 case in India was reported in January 2020 which quickly spread all over the country (Supplementary Figure S1). By the end of December 2021, the total infected population in India was above 34.79 million, while more than 479 thousand people have succumbed to the disease (https://covid19.who.int/table) (Supplementary Table S1). Lack of precise antiviral drugs is a major challenge in restricting the spread of the virus. The development and the increased use of safe vaccines may prove promising in future eradication of SARS-CoV-2^2^. However, due to continuous mutation of RNA viruses including SARS-CoV-2, the efficacy of vaccines against the newly derived lineages than that of reference genome (against which the vaccines were designed) is a matter of concern^3^. Genomic and epidemiological surveillance have been seen as a gold standard for control of contagious diseases. It helps in containing the transmission of the virus by identifying different novel viral variants^2^.

As the COVID-19 cases in India increased the Ministry of Health and Family welfare (MoHFW), Council of Scientific and Industrial Research (CSIR), Department of Biotechnology (DBT) and Indian Council of Medical Research (ICMR) established the Indian SARS-CoV-2 Genomics Consortium (INSACOG) (https://dbtindia.gov.in/insacog) on December 2020 for genome surveillance of the circulating SARS-CoV-2 variants. The consortium initially comprised of 10 national laboratories situated in different states in India acting as hub INSACOG genome sequencing labs (IGSL). For smooth distribution of viral samples and genomic surveillance, later, many more laboratories joined as satellite INSACOG genome sequencing laboratories (https://www.mohfw.gov.in/pdf/INSACOGGuidanceDocumentdated15July2021final.pdf) across the country,studying on the variants of concerns/interests (VoCs/VoIs). It certainly mitigates the COVID-19 cases. The genomic surveillance strategy focuses on sequencing of viral samples from sentinel regions, surge sites and international travellers arriving in India. This is followed by deposition of sequencing data, eventual collation and integration of data by National Centre for Disease Control (NCDC). Decentralized data collection at NCDC is done by Central Surveillance Unit (CSU) and Integrated Disease Surveillance Programme (IDSP). Finally, the data interpretation and analysis is done to correlate genome variants with epidemiological or clinical trends to track unusual events such as suspected reinfection, super spreader, vaccine breakthrough, and epidemic outbreaks of SARS-CoV-2 (https://www.mohfw.gov.in/pdf/INSACOGGuidanceDocumentdated15July2021final.pdf). As of September 2021, around 45,000 SARS-CoV-2 whole genome samples have been sequenced and deposited at GISAID (https://gisaid.org/submission-tracker-global/) by INSACOG laboratories across India.

The vaccines were unavailable during the early phase of COVID-19 in 2020 (Supplementary Figure S2). The Government of India had implemented the process of lockdown, social distancing and use of masks to contain the spread of the deadly virus. The first phase lockdown was announced for a period of 21 days on March 24, 2020. Based on the success of the first round of lockdown, several phases of lock-downs were implemented spanning around 14 days each. India remained in nation-wide lockdown mode until May 31, 2020. It was quite evident that the lockdowns helped in curbing or restrict COVID-19 new cases in India (Supplementary Figure S1). Next, lockdown was imposed in the confined containment zones, while rest parts of the country were allowed to reopen gradually. Along with lockdowns government also emphasized on public awareness related to social distancing, hand washing and use of face masks^4^. The infected individuals were mandatory put under quarantine for curbing the spread of further infection. In India, mass immunization against SARS-CoV-2 was started on January 16, 2021 using Oxford-AstraZeneca vaccine (licensed as Covishield) and Bharat Biotech’s Covaxin^5^ (Supplementary Table S2). With time several other vaccines (Sputnik V from Dr. Reddy’s and ZyCoV-D from Zydus Cadila) became available^5^. The vaccination drive carried out in 2021 saved the lives of 4.2 million people and the vaccination is still ongoing with a view to immunize 100% population in India^6^. During the peak of second COVID-19 wave (May-June, 2021), number of people vaccinated (with at least 1 dose) was found to be less than 10% of total population in India. A detailed overview of the vaccination status in India in 2021 is shown in Supplementary Figure  S2. Despite immunization via vaccination or acquiring protection via natural infection, reinfections were observed in COVID-19^7^. It might possibly due to mutations of SARS-CoV-2, along with the durability of natural and vaccine-mediated immunity^8^.

SARS-CoV-2 acquire genetic mutations similar to other RNA viruses^2^ and these alterations lead to genesis of more communicable variants or variants of concern (VoC). Genome analysis of SARS-CoV-2 revealed that the Spike protein including Receptor Binding Domain (RBD) of homo-trimeric spike glycoprotein are altered in these communicable VoCs. These alterations are responsible for the spread as well as for prolong COVID-19. The RBD of Spike protein participates in attachment to host cell ACE-2 receptor, thereby triggering an array of reactions for viral entry into the host cell^9^. Mutations in the RBD region are found to be responsible for increased activity of ACE-2^10^, subsequently leading to a massive surge in the infection rates.

Here, we aimed at understanding the evolution of different variants of SARS-CoV-2 in different parts of India during the onset and subsidence of second wave (January–September, 2021) using genome based data. Owing to the sudden emergence of B.1.1.529/BA.* (Omicron-like; first detected in Hong Kong/South Africa) variant, and few cases registered in India in late November 2021 or early December 2021, it is also briefly included in our mutation related (amino acid changes in Spike protein) studies. The amino acid changes of Spike protein of Delta, Delta-like and Omicron-like and its’ influence in host-receptor binding and antigenicity has also been discussed.

## Results

### COVID-19 cases and associated deaths across the world in 2021

**Figure 1:**
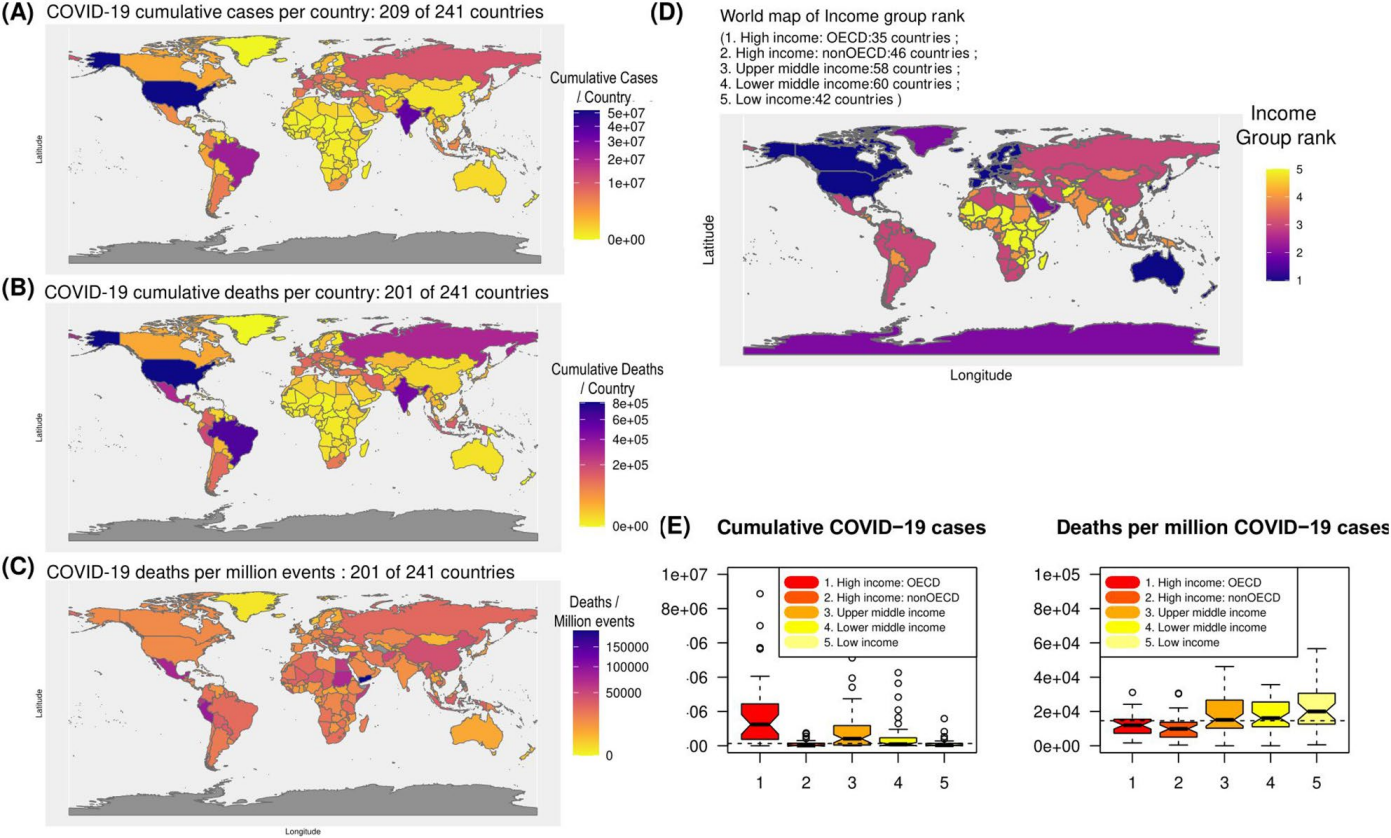
COVID-19 in world scenario in 2021. (**A**) Cumulative cases of COVID-19 per country, (**B**) cumulative COVID-19 associated deaths per country, (**C**) cumulative deaths per million events (COVID-19) per country and (**D**) income group rank of the country is plotted in world map. (**E**) Box plot of COVID-19 cumulative cases or deaths with respect to income group ranking of the countries. The boundaries or geographical coordinates are used only for data representation. The administrative/geographical boundaries might differ. The spatial polygons or points for world map is obtained using R package (rnaturalearth)^11^.

There are 279,114,972 ($\approx$ 279 million) cumulative COVID-19 cases across the world population (https://covid19.who.int/table) in 2021. The cumulative deaths associated with COVID-19 are 5,397,580 accounting on an average 19,338 (1.93%) deaths per million COVID-19 cases. In India, 13,797 (1.34%) deaths per million COVID-19 cases was reported, whereas, 15,643 (1.56 %) deaths per million cases was reported in the United States of America (USA). The details on total cumulative cases, cumulative deaths or deaths per million cases are shown in Fig. 1A–C, and Supplementary Table S1).

Based on cumulative cases as of December 2021, USA, India and Brazil have reported the highest number of cases both in terms of cumulative cases and cumulative deaths. Interestingly, the deaths per million reported cases are found to be more in low income and lower-middle income countries like Yemen, Sudan, Peru, Mexico, Syria, etc. Box plot clearly indicates that cumulative COVID-19 cases are more in developed nation with high-income group. It might be due to developed infrastructure and testing centers for COVID-19. Although the reported cases are lower in low-income countries, but deaths associated with COVID-19 are relatively higher in poor nations (Fig. 1D,E). It might be due to lesser testing centers, poor medical facilities and social awareness. India, the country with estimated population of  $1.3 \times 10^{9}$, and falling under category 4; lower-middle income is expected to be affected by pandemic COVID-19 as shown in Fig. 1. Here, we focus on India, which has faced the catastrophic consequences of the second wave (April–June 2021) with peak during the month of May 2021. The distribution of incidences and deaths related to COVID-19 in India in 2021 also depicts the peak in May (Supplementary Figure S1).

### Genome surveillance of SARS-CoV-2 in 2021 in India

**Figure 2:**
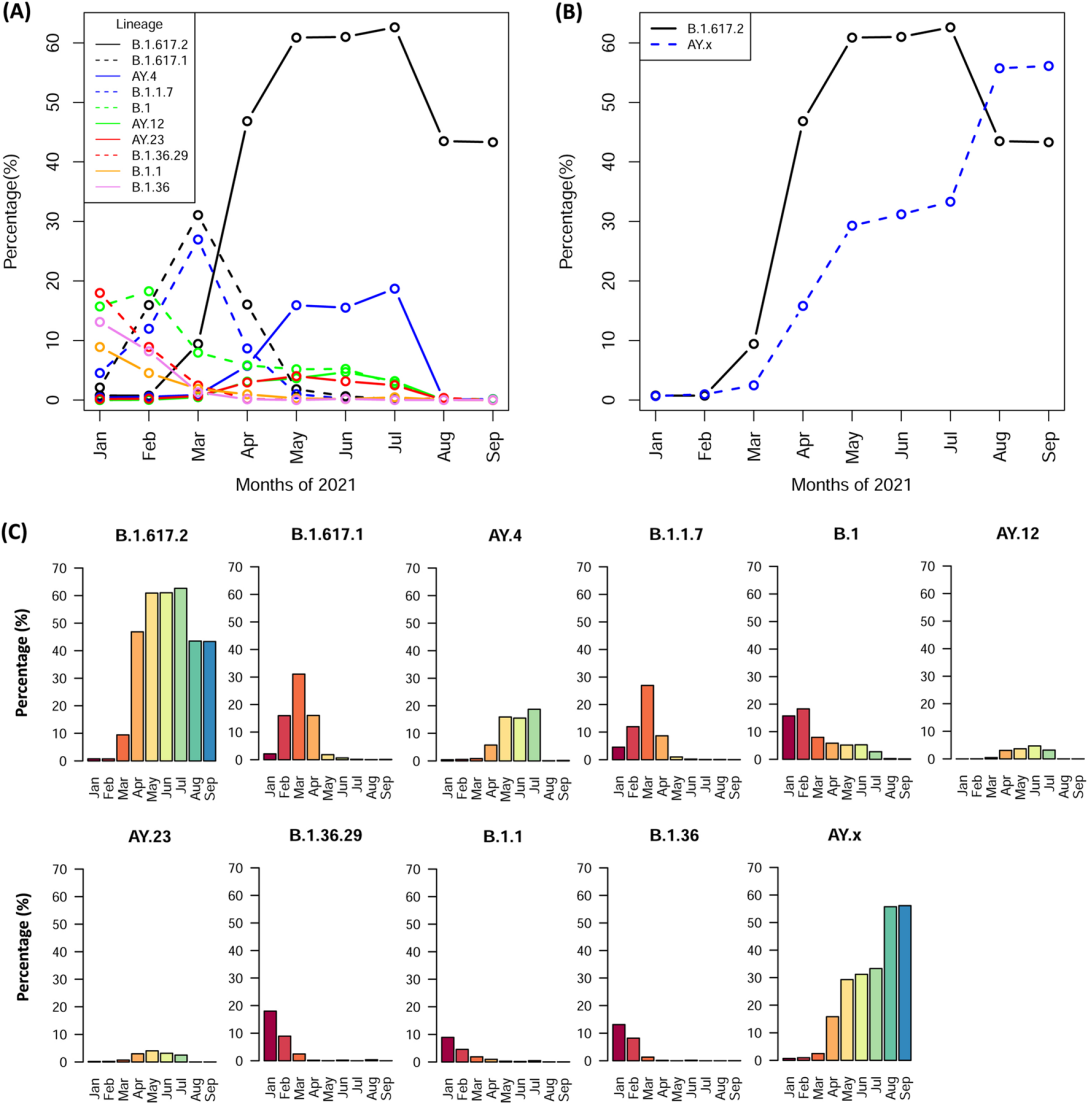
Genome Surveillance of top 10 VoCs in India during Jan–Sept 2021. (*A,B*) Scatter plot of incidences of various lineages as indicated per month. (**C**) Bar plot of respective lineages as indicated.

To study the details and the possible VoCs of SARS-CoV-2 prevalent in India during the catastrophic second wave, we retrieved patient metadata and the associated genome sequences (n = 44514) from GISAID for the month of January (4 months prior second wave) to September, 2021 (4 months post second wave). The respective metadata file (Supplementary Table S3) of all VOCs and VoIs as described in the Methodology section were selected for the analysis. At least 38 SARS-CoV-2 VoCs/VoIs are found across India from January to September 2021 with minimum 100 recorded cases (Supplementary Table S4). The lineages (VoCs/VoIs) were ordered in descending manner based on incidences, and top ten different variants of the virus as identified in India till September 2021 are plotted (Fig. 2). Due to various sub-lineages of B.1.617.2 (Delta), AY.* (Delta-like), subseries was continuously renamed by Pango nomenclatures in GISAID. AY.4 got reclassified as AY.4.2, AY.12, AY.22, AY.23, AY.103, AY.122, etc. Hence, a generalized term ’AY.x’ is being used for the newly classified AY.4 variant. The graph is constructed based on percentage of incidences of VoCs in the respective month. The top ten lineages found based on their prevalence (in %) per month are shown in Table 1 and Fig. 2.

An analysis of the plot depicts variants namely B.1, B.1.1, B.1.17, B.1.36 and B.1.36.29 were prevalent in the month of January. Likewise, a similar pattern of prevalence except a rise in B.1 cases was observed in February. In March, a decline of all the other lineages could be seen while rise in the B.1.17, B.1.617.1 and B.1.617.2 (aka Delta variant) were reported. B.1, B.1.1.7, and B.1.617.1 remained prevalent prior to the second wave in India (Fig. 2, Supplementary Figure S3). B.1617.2 surge/peak was found in the month of May that coincides with the time of the second wave in India. In due course of time, B.1.617.2 gained more mutations that gave rise to sub-lineages of Delta-like, namely, AY.x. Both Delta/Delta-like constitutes more than 90 % of load of all cases per month during the peak and thereafter (Table 1, Fig. 2B). During the post second wave, all other lineages including Kappa, which was prevalent during the onset or prior to the second wave slowly diminished and were unable to compete with the Delta/Delta-like lineages.

### B.1.617.2 (Delta) and AY.x (Delta like) distribution in Indian states and territories

**Figure 3:**
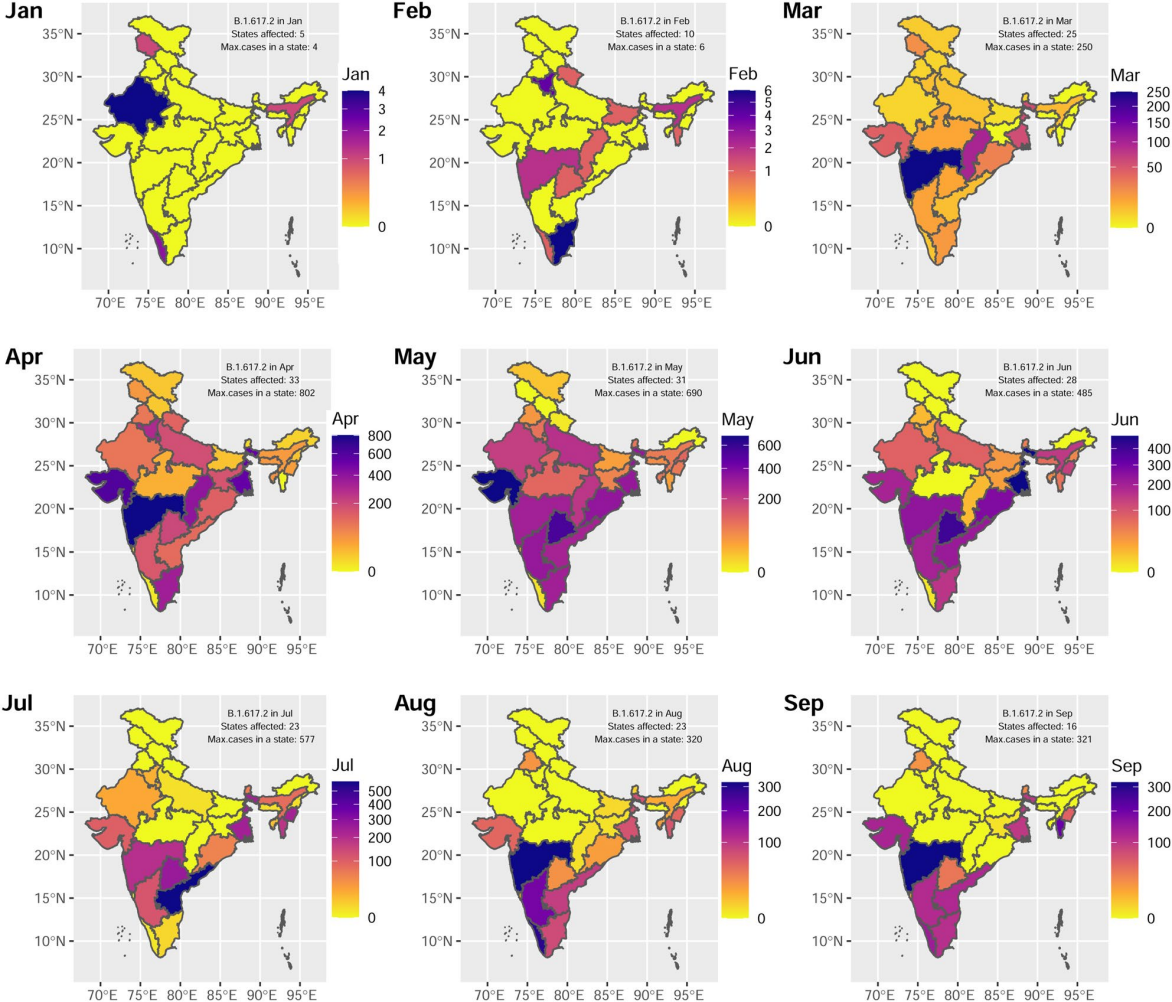
Spatiotemporal transmission/incidences of B.1617.2 (Delta) in Indian states and union territories (UTs). The dynamic incidences of Delta-surge in Indian states and UTs were shown in indicated months (**A–I**). The gradient scale showed the number of events/incidences of Delta for different states and UTs. The geographical/administrative boundaries are used only for the purpose of data representation. The spatial polygonal coordinates or boundaries of the states and UTs of India are obtained from the link url: https://gadm.org/download_country.html selecting the country India and the shape file: R (sf), level1.

In order to study the kinetics or virus spread which is mainly contributed by Delta lineage, dynamic distribution of B.1.617.2 in different states and union territories of India during January to September, 2021 is plotted (Fig. 3). The map shows that in January, only five (5) Indian states reported B.1.617.2 cases that incresed to 31 affected states during the peak (May) of the second wave based on the reported cases in GISAID. From July onwards, northern and central states of India reported a decline in B.1.617.2 cases while the case load borne by B.1.617.2 remained almost the same in the Indian southern states. Along with the Delta variant, a hike in incidences of AY.4/AY.x (aka Delta plus) was also eminent. AY.4/AY.x followed the similar pattern as B.1.617.2 but with lesser incidences (% cases = 13.98 ± 4.01) in peak periods (Fig. 2, Table  1, Supplementary Figure S4), but reached more than 50% of incidences by the month of September.

### Correlation of different SARS-CoV-2 lineages and states

**Figure 4:**
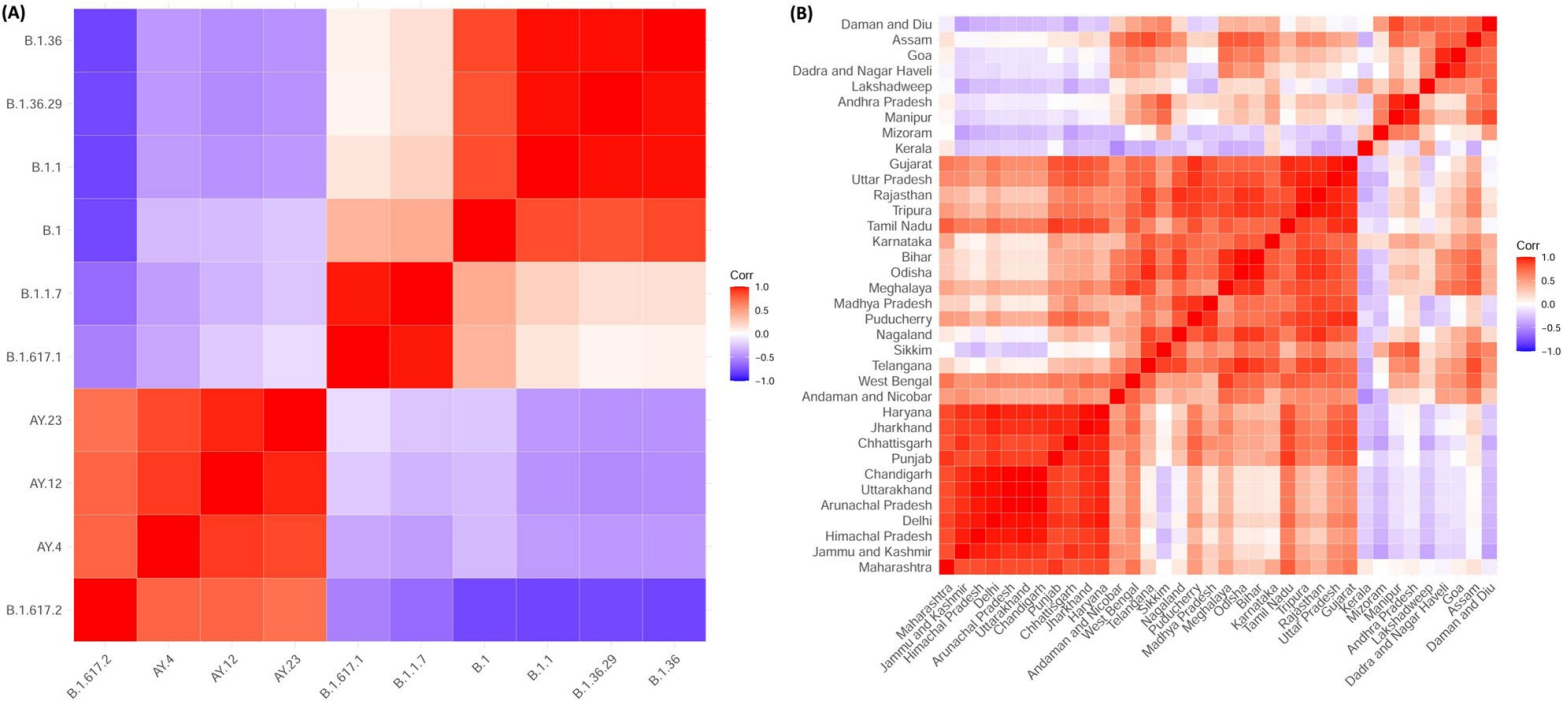
Hierarchical clustering of correlation matrix of various lineages. (**A**) Correlation plot of (**A**) different variants of SARS-CoV-2 lineages found in Indian population. (**B**) Correlation plot different states/UTs with respect to cases of Delta VoC lineage obtained per month.

A correlation analysis of the VoCs/VoIs was done based on the incidence percentage (%) per month of different variants of SARS-CoV-2. Here we have studied only the top 10 lineages based on cumulative cases of the variants during January–September 2021. The matrix was plotted on a − 1 to + 1 scale where positive correlation ($>0$) and negative correlation ($<0$) are shown in gradient scale. Zero (0) denotes no correlation. Hierarchical clustering was performed between various VoCs or lineages (Fig. 4A). The analysis shows that B.1.617.2 has a high to moderate correlation (0.5–1) with AY.4, AY.12 and AY.23 based on incidences per month. This also indicates the coexistence of Delta and Delta-like, together. On the other hand, Delta and Delta-like show a negative correlation with other variants (B.1, B.1.1, B.1.1.7, B.1.36, B.1.362.9 and B.1.617.1). Interestingly, B.1.617.1 and B.1.17 are highly correlated, and are clustered together, but both shows moderate correlation with B.1. The third distinguished cluster formed by B.1.36, B.1.36.29 and B.1.1 are all positively correlated among themselves, but most distantly correlate with Delta and Delta-like VoC. The correlation pattern highlights that the Delta and its different sub-lineages (B.1.617.2, AY.4, and AY.23) peak periods/phases are similar and inversely related with all other VoCs/VoIs based on incidences.

In India, the Delta-surge is the main reason of catastrophic COVID-19 associated deaths. Hence, we have studied the kinetics of Delta-cases associated with the respective states of India (Fig.  3). To know if there is a similar pattern of Delta-surge across the country, we have performed a correlation analysis followed by hierarchical clustering (Fig.  4B).There is moderate to high similarity among the cluster states. Based on K = 2, we found two clusters as shown in Supplementary Figure S5. The majority of the states fall in cluster-1 whereas cluster-2 is formed by states/UTs comprising Mizoram, Kerala, Dadra and Nagar Haveli, Goa, Lakshadeep, Manipur, Andhra Pradesh, Assam, and Sikkim. These states/UTs of cluster-2 are distantly related from cluster-1 (Supplementary Figure S5) and are possibly less affected by Delta-wave in India (Fig.  3).

### Amino acid changes (substitutions/deletions) within the Spike protein of the different variants

**Figure 5:**
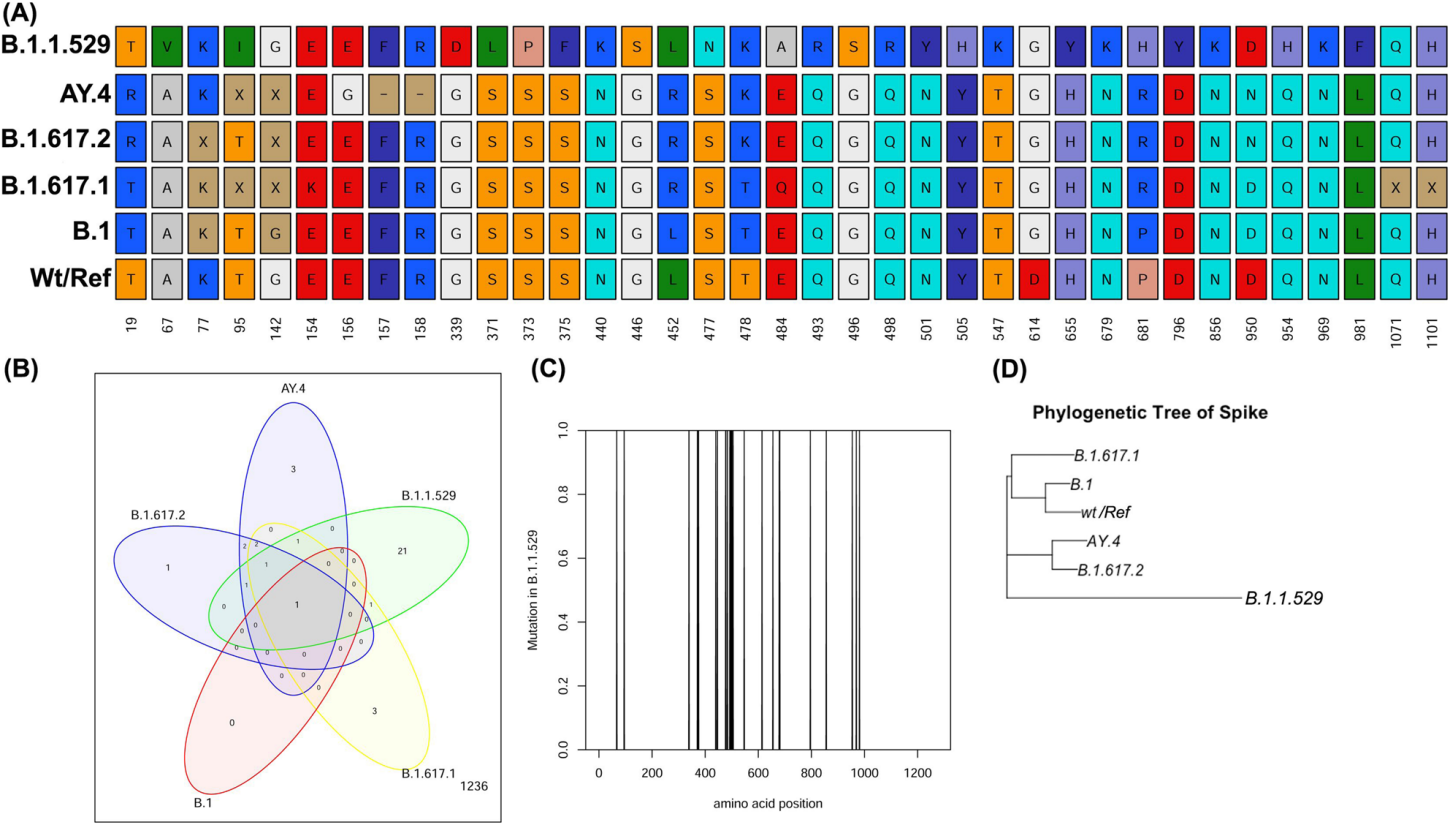
Mutations in various VoC circulating in Indian population in Jan to Sept 2021. (**A**) Mutation in amino acids of various VoCs with respect to wild type/Reference (wt/Ref) S protein is shown along with the position as indicated in number. (**B**) Venn diagram showing overlapping and unique mutations among various VoCs. (**C**) Enriched mutational sites of Omicron (B.1.1.529) lying in the S protein. (**D**) Phylogenetic tree of S protein of various VoCs as indicated.

In order to study whether the lineages are associated with the viral spread^12^ and rise in the COVID-19 cases, we further consolidate our findings in mutations in the RBD of spike protein (S) (Fig. 5A). The spike protein consists of 1273 amino acids. The amino acids lying on the position 319–541 constitutes/forms the RBD region^13^. B.1.617.2, AY.4/AY.x, B.1.617.1, B.1, B.1.1.529 (Omicron) and the NC_045512 (Wild type/Reference) denoted as Wt/Ref were selected for the analysis. Fifty genome sequences of each strain having genome length of > 29,000 were used to obtain consensus nucleotide sequence of each lineage or VoC. The consensus nucleotide sequence was further translated to obtain amino acid sequences of the Spike protein. Multiple sequence alignment of all the lineages/VoCs was done against the reference strain using R package ‘msa’ and the changes/substitutions against each amino acid was identified. Major amino acid changes or substitutions are observed within 19–950 region of the S protein. From the analysis, it is observed that B.1 and Wt/Ref has similar S protein sequences except D614G mutation. All the other variants also shows the similar change at D614G. The variants B.1.617.1, B.1.617.2 and AY.4/AY.x shows a significant L452R mutation in RBD site and another P681R substitution. Interestingly, B.1.617.2 and AY.4/AY.x shows another signature T478K substitution in the RBD region. Moreover, both the variants (B.1.617/2/AY.x) also has a unique D950N mutation. The E156G mutation is unique to AY.4, along with two deletions of amino acids at amino acid position 157 and 158 with respect to the Reference S protein. The mutation analysis further highlights a completely different architecture of Omicron with 21 signature mutations (unique mutations) as shown by Venn diagram (Fig.  5A,B). The mutations of Omicron is enriched in the RBD region of the S protein (Fig. 5C).

To study the phylogenetic relationship, pairwise distances from aligned sequences were computed using identity matrix. Neighbor-joining method^14^ was applied on these obtained identity matrix. The topology of the tree reveals similarity of S protein between the Reference(NC_044512) and B.1, followed by their close relatedness with B.1.617.1. The Delta (B.1.617.2) and Delta-like (AY.4) fall under different cluster and are more closely related among themselves. Due to extensive mutations in Omicron Spike protein, it belongs to a separate cluster (Fig. 5D).

### Amino acid changes/substitutions in spike (S) protein on SARS-CoV-2 affecting its interaction with ACE-2 receptor and antigenicity

To elucidate the phenotypic effect of amino acid mutations with host cell binding, we performed GISAID enabled CoVserver analysis (Table 2) on Delta/Delta-like VoC along with the emerging Omicron. Delta/Delta-like variants show mutations (L452R, T478K, D614G, P681R) that possibly play a role in host-cell interaction and in antigenicity. Moreover, the entropy was found to be positive (Table 2) indicating that the variant is thermodynamically more stable than wild type strain. This might be the possible reason of sudden surge in Delta/Delta like VoCs as compared to wild type or more closely related VoCs. Interestingly, Omicron, an emerging VoC shows a maximum number of amino acid changes in the Spike protein enriched in the RBD and its periphery region (G339D, S371L, S373P, N440K, G446S, S477N, T478K, E484A, Q493R, G496S, Q498R, N501Y, Y505H, D614G, and P681H) (Table 2), as compared to wild type and Delta and Delta-like VoCs. Majorly, these amino acid positions participate in the host cell ACE-2 binding and antibody recognition. Apart, they also help in ligand binding and viral oligomerization^15^. The high positive entropy change ($\Delta$ S = 26) for Omicron as compared to reference strain (hCoV-19/Wuhan/WIV04/2019) suggests that it can be energetically more stable and expected to outnumber Delta in the near future. The presence of high number of mutations in the Omicron predicts that it could be the next VoC potentially responsible for massive surge in COVID cases in India.

## Discussion

The sudden surge of COVID-19 cases in India in 2021 and the devastating second wave corresponds with the prevalence of B.1.617.2 (aka Delta variant) and its other dominant sub-lineages AY.4/AY.x^16^. Increased incidences of Delta could be linked to its high transmissibility, ability to evade immune responses within human body and diagnostic detection failure^17^. High density of population is directly proportional to increased viral replication, mutation and evolution. Such evolutionary processes cause generation of more transmissible and pathogenic viral mutants^18^. Dense population of India and poor containment strategies are one of the driving forces behind the deadly second wave. The decline in B.1.617.2 in northern states of India during post second wave may be attributed to herd immunity as those states/UTs are affected mostly during the peak of the second wave in India.

Apart from epidemiological considerations, the prime cause of Coronavirus transmission and pathogenicity is underlain in its structure. A comprehensive overview of the SARS-CoV-2 structure reveals presence of single stranded positive sense 29.9 kb RNA genome^19^. It contains four structural proteins viz. spike (S), envelope (E), membrane (M) and nucleocapsid (N). Apart from structural proteins, 16 non-structural proteins (nsp 1–16) help in different viral processing^20^. Among all the structural proteins, spike protein is of special research interest because it mediates host cell attachment and entry^20,21^. The detailed structural analysis of homotrimeric S glycoprotein reveals the presence of the two functional subunits S1 and S2. The S1 contains receptor binding domain (RBD) helping in binding of the virion particle to host cell receptor such as ACE-2^22^. The S2 subunit functions in fusion of virus and host cell membranes^20^. Our observations on unprecedented surge of Delta (B.1.617.2) and Delta plus (AY.4/AY.x) in India could be attributed to its unique mutations within the spike protein. The mutation D614G, which was observed in all SARS-CoV-2 variants, was reported to be one of the most predominant mutation^23^. Earlier studies have suggested that this D614G mutation provides a certain replication advantage to the virus. Thus, D614G was associated to increased human transmission of SARS-CoV-2 infections^24–26^. The spike protein mutation (L452R) which is found in B.1.617.1, B.1.617.2 and AY.4/AY.x is very important for the pathogenicity of the virus. It confers increased ACE-2 binding affinity and decreased antibody binding capacity, thereby increasing host immune evasion by the virus^27,28^. The mutation (P681R) has been reported to facilitate S1–S2 cleavage at Furin cleavage site^27^. All these mutations facilitate viral replication, transmission and virulence within the host cells. Therefore, our findings on predominance of B.1.617.1, B.1.617.2 and AY.4/AY.x over other variants corroborated well with the presence of these mutations within them. Some of the signature mutations of Delta variants are T19R, T478K and D950N (Fig. 5), which are present in the N terminal domain, RBD and S2 region respectively. Previous literature has highlighted that T19R and T478K (located in epitope binding region) impairs monoclonal antibody mediated neutralization while D950N affects spike protein dynamics thereby increasing the virulence of SARS-CoV-2^29^. So, B.1.617.2 and AY.4/AY.x out-competed or displaced B.1.617.1 as well as other variants possibly by evading innate or vaccine induced immunity. In November 2021, a new variant Omicron (B.1.1.529, BA.1) appeared with 21 signature and 5 overlapping mutations within its spike protein (Fig. 5). The changes in the spike protein of Omicron can be linked to its increased virulence so surveillance of Omicron or Omicron-like VoCs will be very important for the coming days. Since our study mainly deals with the genomic surveillance of SARS-CoV-2 during January to September, 2021 (during onset, peak and post second wave of COVID-19 in India), Omicron is excluded in our surveillance analysis due to lack in incidences of Omicron during this period. However, considering the latest scenario of Omicron or Omicron-like VoC surge in several countries including South Africa, UK, USA, etc., tracking of Omicron is needed. Due to its high mutations (around 30 amino acids changes) within the spike protein, the mutations of S protein of Omicon-like VoC were analyzed for its role in host-cell receptor binding and antigenicity. There were almost 10–15 (Table 2) amino acids falling in the RBD and its periphery region, influencing the interactions and antigenicity. Although people in India is immunized against SARS-CoV2 either via mode of vaccination or by herd immunity, the mutations of amino acids might provide an adaptive advantage to overcome immune response or escape antibody neutralization and hence, surge in Omicron is anticipated in India too.

**Table 1:** Incidences of reported cases of various VoCs per month.

Month	B.1.617.2	B.1.617.1	AY.4	B.1.1.7	B.1	AY.12	AY.23	B.1.36.29	B.1.1	B.1.36	AY.x	B.1.617.2/AY.x
Jan	0.76	2.1	0.42	4.54	15.71	0	0.17	17.98	8.91	13.11	0.67	1.43
Feb	0.74	15.95	0.55	11.97	18.28	0.04	0.22	8.92	4.53	8.18	0.96	1.69
Mar	9.43	31.07	0.84	26.96	7.96	0.47	0.67	2.44	1.88	1.31	2.47	11.9
Apr	46.85	16.04	5.74	8.67	5.83	3.09	2.95	0.18	0.96	0.12	15.81	62.66
May	60.89	1.82	15.92	0.99	5.17	3.66	4.02	0.04	0.29	0	29.28	90.17
Jun	61.01	0.64	15.52	0.22	5.23	4.69	3.15	0.2	0.16	0.18	31.19	92.2
Jul	62.62	0.14	18.71	0.06	2.75	3.18	2.49	0	0.46	0	33.31	95.93
Aug	43.49	0.03	0.06	0.06	0.22	0	0	0.35	0.03	0	55.74	99.23
Sep	43.31	0.09	0.15	0.03	0.12	0	0	0	0.03	0.03	56.13	99.44

The number denotes the percentage incidences of respective Voc with respect to total incidences of the indicated months.

**Table 2:** Amino acids involved in phenotypic effects of VoCs as compared to reference.

VoC	$\Delta$S	Amino acids for host cell interactions and antigenicity
B.1.1.529	26	G339D, S371L, S373P, N440K, G446S, S477N, T478K, E484A, Q493R, G496S, Q498R, N501Y, Y505H, D614G, P681H
AY.4	11	L452R, T478K, D614G, P681R
B.1.617.2	8	L452R, T478K, D614G, P681R

Genome surveillance of SARS-CoV-2 associated COVID-19 cases in human population in India shows varying distribution of lineages (VoCs/VoIs) in pre, during and post second wave of COVID-19 surge in India in the year of 2021. It was evident that the variant Delta (B.1.617.2) played a major role in the increased COVID-19 cases, and a probable reason of the second wave in India. The spatiotemporal dynamic transmission/incidences of the Delta or Delta-like VoCs in India are reported for the first time by our group. It indicates that the Delta-surge in the different states and union territories of India differs. The north-eastern states and the islands of Indian were less affected by the second wave. Considering the recent surge in Omicron-like VoC, and its possible role in altering the host-cell interactions and antigenicity, it will be noteworthy to conduct genome surveillance focusing on Omicron-like VoC in parallel with Delta-like VoC. It will help in avoiding the stress on the medical systems resulting in fatal consequences due to sudden outburst of incidences of the repetitive waves of COVID-19.

## Methods

### Data mining and plotting

The SARS-CoV-2 genome sequences and patient metadata (n = 44,514) were retrieved from GISAID SARS-CoV-2 database. The entries with complete collection date is selected/ticked while downloading the patient metadata files. The GISAID EpiCoV accession number is attached in Supplementary Table S3. The metafile of GISAID with accession number is attached as supplementary. The nucleotide FASTA files were downloaded and used for the different analyses as mentioned in detailed in the Supplementary Section.

### Spatiotemporal transmission/incidences of Delta in India

The spatial polygonal regions of different states and union territories (UTs) as obtained were described in detailed in the supplementary methods. The metadata file was obtained from GISAID databases for B.1.617.2 (Delta), and per month data is analysed with respect to different states and UTs and is plotted using R/R studio^30^ with ggplot2^31^ and relevant packages^32^. The R code can be shared on request.

## Supplementary Information

Supplementary Information 1.

Supplementary Information 2.

Supplementary Information 3.

Supplementary Information 4.

Supplementary Information 5.

## Data Availability

The relevant data and the supplementary files are shared in the manuscript along with GISAID EpiCoV accession number. The GISAID EpiCoV accession number is attached in Supplementary Table S3. The code for R/Rstudio^11^ used for plotting and data analysis can be found from the link: https://rpubs.com/siddik/GenomeSurveillance or by sending request to corresponding author: siddik.sarkar@iicb.res.in.

## References

[1] Hennekens CH, George S, Adirim TA, Johnson H, Maki DG (2020). The emerging pandemic of coronavirus and the urgent need for public health leadership. Am. J. Med..

[2] Robishaw JD (2021). Genomic surveillance to combat covid-19: Challenges and opportunities. Lancet Microbe.

[3] LopezBernal J (2021). Effectiveness of covid-19 vaccines against the b.1.617.2 (delta) variant. N. Engl. J. Med..

[4] Krishan K, Kanchan T (2020). Lockdown is an effective ‘vaccine’ against covid-19: A message from India. J. Infect. Dev. Ctries..

[5] Chakraborty C, Sharma AR, Bhattacharya M, Agoramoorthy G, Lee S-S (2021). The current second wave and covid-19 vaccination status in India. Brain Behav. Immun..

[6] Watson OJ (2022). Global impact of the first year of covid-19 vaccination: A mathematical modelling study. Lancet Infect. Dis..

[7] Wang J, Kaperak C, Sato T, Sakuraba A (2021). Covid-19 reinfection: A rapid systematic review of case reports and case series. J. Investig. Med..

[8] Townsend JP, Hassler HB, Sah P, Galvani AP, Dornburg A (2022). The durability of natural infection and vaccine-induced immunity against future infection by SARS-CoV-2. Proc. Natl. Acad. Sci. USA.

[9] Letko M, Marzi A, Munster V (2020). Functional assessment of cell entry and receptor usage for sars-cov-2 and other lineage b betacoronaviruses. Nat. Microbiol..

[10] Wan Y (2020). Receptor recognition by the novel coronavirus from Wuhan: An analysis based on decade-long structural studies of SARS coronavirus. J. Virol..

[11] South, A. rnaturalearth: World Map Data from Natural Earth (2017). R package version 0.1.0. https://CRAN.R-project.org/package=rnaturalearth.

[12] Saha P (2020). Mutations in spike protein of sars-cov-2 modulate receptor binding, membrane fusion and immunogenicity: An insight into viral tropism and pathogenesis of covid-19. ChemRxiv.

[13] Huang Y, Yang C, Xu X-F, Xu W, Liu S-W (2020). Structural and functional properties of sars-cov-2 spike protein: Potential antivirus drug development for covid-19. Acta Pharmacol. Sin..

[14] Saitou N, Nei M (1987). The neighbor-joining method: A new method for reconstructing phylogenetic trees. Mol. Biol. Evol..

[15] Starr TN (2020). Deep mutational scanning of sars-cov-2 receptor binding domain reveals constraints on folding and ace2 binding. Cell.

[16] Tareq AM, Emran TB, Dhama K, Dhawan M, Tallei TE (2021). Impact of sars-cov-2 delta variant (b.1.617.2) in surging second wave of covid-19 and efficacy of vaccines in tackling the ongoing pandemic. Human Vaccines.

[17] Singh J, Rahman SA, Ehtesham NZ, Hira S, Hasnain SE (2021). Sars-cov-2 variants of concern are emerging in India. Nat. Med..

[18] Asrani P, Eapen MS, Hassan MI, Sohal SS (2021). Implications of the second wave of covid-19 in India. Lancet Respir. Med..

[19] Lu R (2020). Genomic characterisation and epidemiology of 2019 novel coronavirus: Implications for virus origins and receptor binding. Lancet.

[20] Wang MY (2020). Sars-cov-2: Structure, biology, and structure-based therapeutics development. Front. Cell. Infect. Microbiol..

[21] Li F (2016). Structure, function, and evolution of coronavirus spike proteins. Annu. Rev. Virol..

[22] Lan J (2020). Structure of the sars-cov-2 spike receptor-binding domain bound to the ace2 receptor. Nature.

[23] Yurkovetskiy L (2020). Structural and functional analysis of the d614g sars-cov-2 spike protein variant. Cell.

[24] Antoneli F, Furuyama T, Carvalho I, Briones M, Janini L (2021). Research article temporal data series and logistic models reveal the dynamics of sars-cov-2 spike protein d614g variant in the covid-19 pandemic. Genet. Mol. Res..

[25] Korber B (2020). Tracking changes in sars-cov-2 spike: Evidence that d614g increases infectivity of the covid-19 virus. Cell.

[26] Volz E (2021). Evaluating the effects of sars-cov-2 spike mutation d614g on transmissibility and pathogenicity. Cell.

[27] Cherian S (2021). Sars-cov-2 spike mutations, l452r, t478k, e484q and p681r, in the second wave of covid-19 in Maharashtra, India. Microorganisms.

[28] Starr TN, Greaney AJ, Dingens AS, Bloom JD (2021). Complete map of sars-cov-2 rbd mutations that escape the monoclonal antibody ly-cov555 and its cocktail with ly-cov016. BioRxiv.

[29] Planas D (2021). Reduced sensitivity of sars-cov-2 variant delta to antibody neutralization. Nature.

[30] R Core Team. R: A Language and Environment for Statistical Computing (R Foundation for Statistical Computing, 2022). https://www.R-project.org.

[31] Wickham, H. ggplot2: Elegant Graphics for Data Analysis (Springer, 2016). https://ggplot2.tidyverse.org.

[32] Sarkar P (2022). Genome characterization, phylogenomic assessment and spatio-temporal dynamics study of highly mutated BA variants from india. Indian J. Med. Microbiol..

