# Supplementary material for: Genome surveillance of SARS-CoV-2 variants and their role in pathogenesis focusing on second wave of COVID-19 in India
Source: Sci Rep. 2023 Mar 22;13:4692. doi: 10.1038/s41598-023-30815-5 (PMC10031706; doi:10.1038/s41598-023-30815-5)
Supplement: Supplementary file 1 — Supplementary Information 1. [file 41598_2023_30815_MOESM1_ESM.pdf]

SUPPLEMENTARY DATA

Methods

Plotting geographical locations/map of the word and India

The spatial polygons or points for world map is obtained using R[1] package (rnaturalearth)[2]. The Indian map for the states are obtained from the link url: https://gadm.org/download_country.html, selecting the country India and the shape file: R (sf), level1. The plotting is done using R package (ggplot2)[1,3,4].The data (table) for COVID-19 cases across the world with days/ months/ year is downloaded from the link: https://covid19.who.int/table.

Plotting of prevalence of SARS-CoV-2 variant data

All the lineages and their prevalence based on incidences in different states in India in 2021 were ordered with the most prevalent at the top and the least prevalent at the bottom. The top ten lineages based on incidences were selected and scatter plot was constructed using the R/RStudio[1].

Correlation Plot construction and visualization

The correlation matrix based on the prevalence pattern of the lineages was plotted using R/Rstudio. Pearson correlation coefficient method was used for calculating the correlations. Hierarchical clustering of correlation matrix and visualizations of correlation plot was done using R/R studio[1] with relevant package ggplot2 [3].

Analysis of amino acid substitutions/changes in the spike protein

The best fifty FASTA nucleotide sequences of genomes of respective SARS CoV-2 variants were downloaded. The selection criteria are: complete genome sequences (>=29000 bases and <1% Ns), high coverage (<1% Ns), exclude low coverage (exclude sequences > 5% Ns) and sequences with entries with complete collection date. The nucleotide sequences were aligned with respect to NCBI Reference Sequence: NC_045512.2 starting at 21563 and extending up to 25384 using R package "msa" to obtain a nucleotide consensus sequence representing various lineages. These spike (S) glycoprotein sequence region (21563-25384) was further translated using R package "Biostrings". The consensus amino acid sequences of the respective lineages were further aligned with amino acid sequences of the Wild type S protein or NCBI Reference Sequence: YP_009724390.1. The aligned sequences were further analyzed for amino acid substitutions/ changes in the different lineages (VoCs/VoIs) with respect to the Wild type S protein.

Supplementary Figures

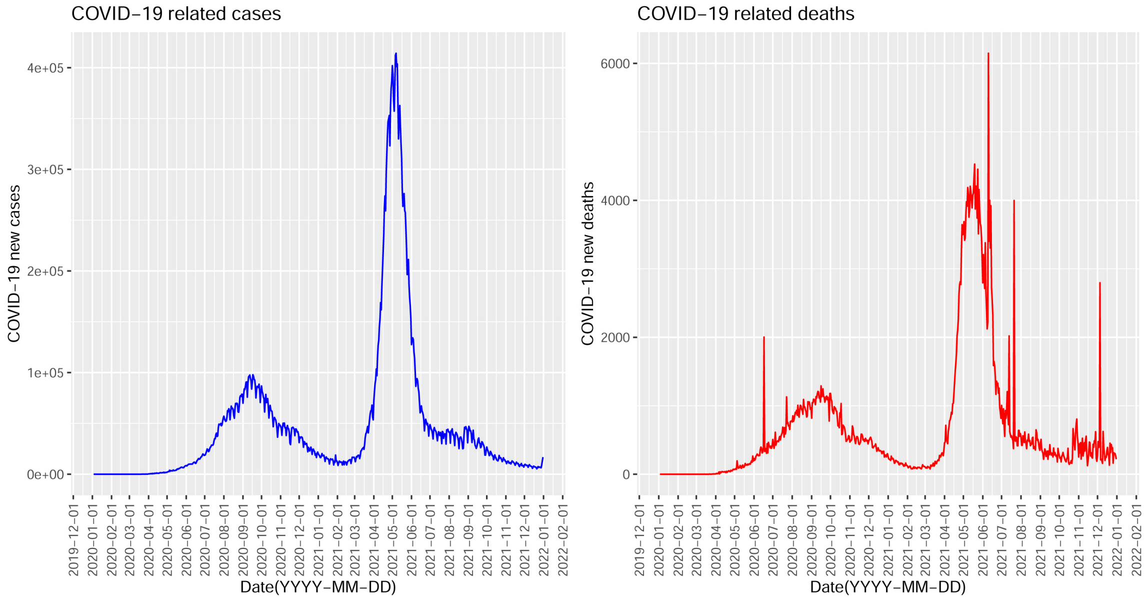

Figure S1. COVID-19 surge/ wave in India

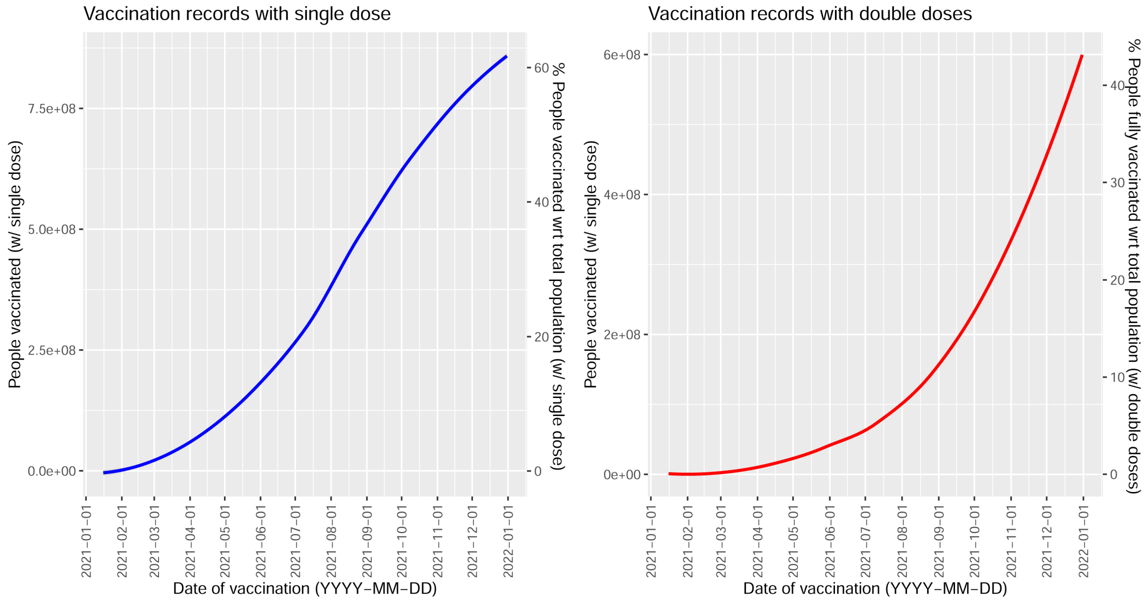

Figure S2. COVID-19 vaccination in India

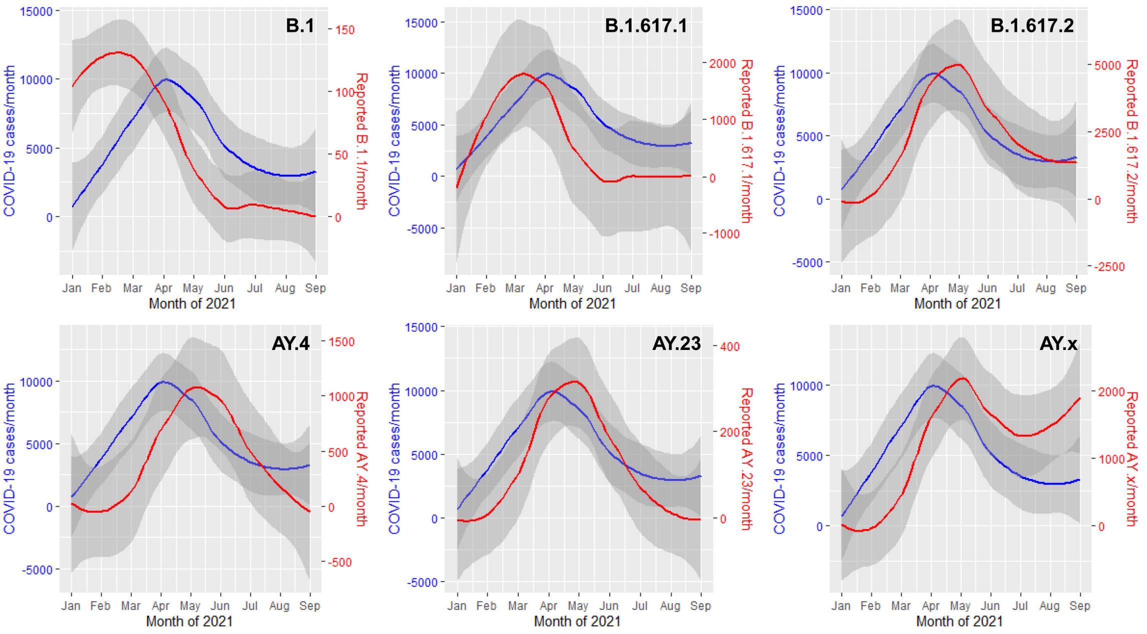

Figure S3. Genome Surveillance of COVID-19 total cases and the respected lineage with respect to months of year 2021. Primary axis (blue colored label) with blue line showing COVID-19 cases observed in the indicated month while secondary y axis (red colored label) with red line indicating reported lineage observed in the indicated month.

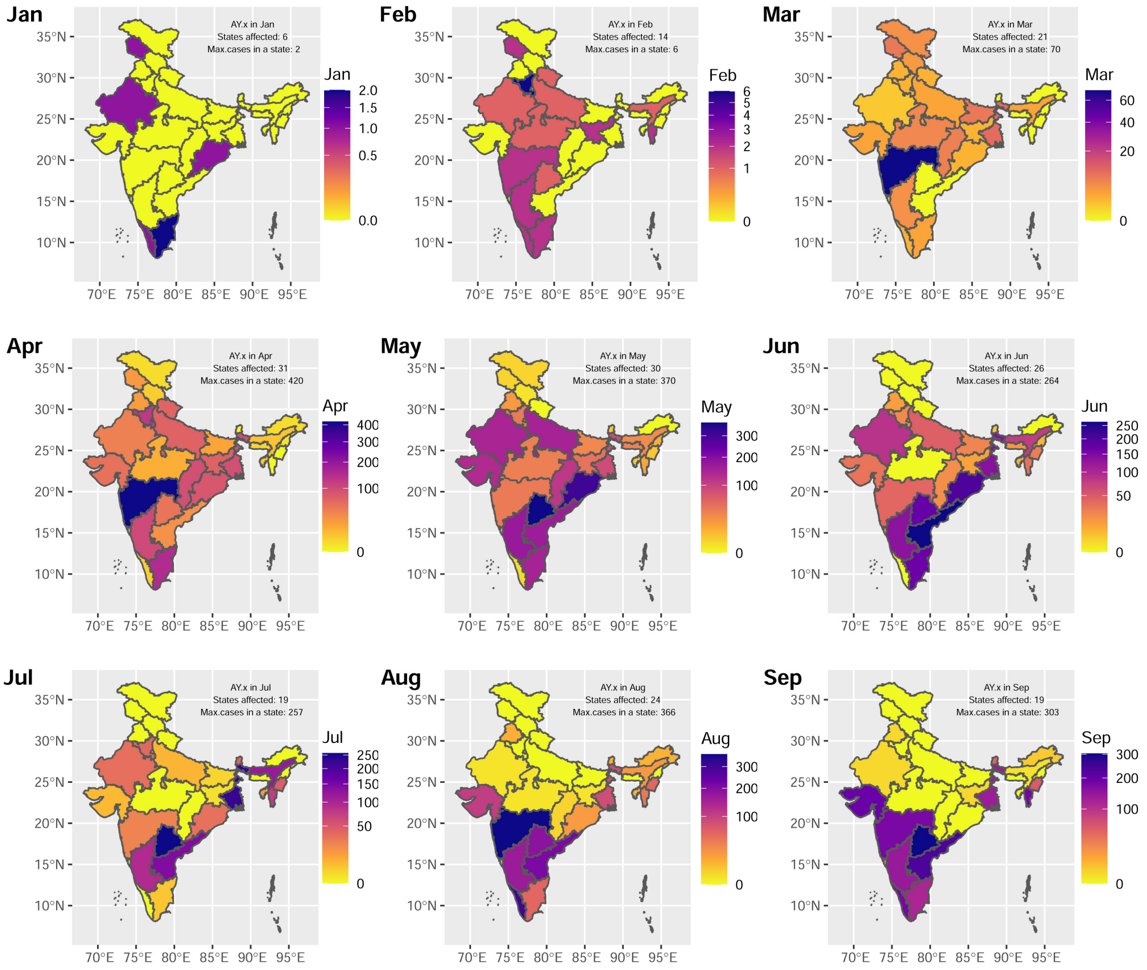

Figure S4. **AY.4/AY.x dynamic distribution in Indian states and union territories (UTs) from the period January 2021 - September 2021**. The reported cases of AY.x (Delta-like) VoC in respective month in different states and UTs of India is plotted. *Note*: The geographical/administrative boundaries might differ. The Indian map for the states are obtained from the link url: https://gadm.org/download_country.html, selecting the country India and the shape file: R (sf), level1.

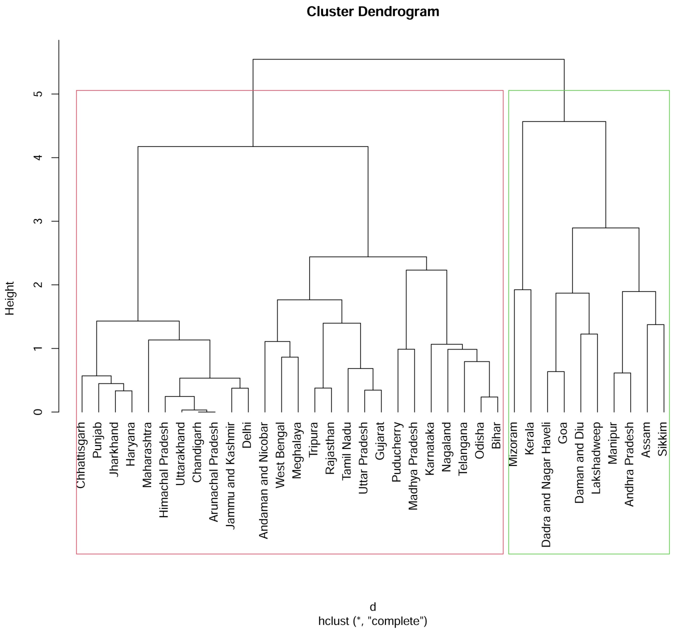

Figure S5. Hierarchical clustering of states /UTs based on delta incidences per month.

Supplementary Tables

Country	Cumulative.cases	Cumulative.deaths	DeathPer10E6Event	income_grp_rank
India	34793333	479997	13795.66	4. Lower middle income
USA	51696204	808701	15643.33	1. High income: OECD
All countries	278154868	5375755	19326.48	NA

Table S1. COVID-19 cumulative cases and death across different countries The Supplementary data with all the countries can be obtained online: Supplementary Table S1.csv

Date of vaccination	Vaccine.name	People vaccinated	Fully vaccinated	link/ sources
16-01-2021	Covaxin, Covishield	191181	0	https://twitter.com/MoHFW_INDIA/status/1350459098203004928
13-02-2021	Covaxin, Covishield	8044786	7668	https://pib.gov.in/PressReleseDetailm.aspx?PRID=1697804
30-09-2021	Covaxin, Covishield, Sputnik V	648949043	237476249	https://dashboard.cowin.gov.in/
31-12-2021	Covaxin, Covishield, Sputnik V	845640601	603224821	https://dashboard.cowin.gov.in/

Table S2. COVID-19 vaccination records in India The Supplementary data with date and vaccination records and other detailed information's can be obtained online: Supplementary Table S2.csv

Virus.name	Accession.ID	Lineage	Collection.month
hCoV-19/India/MH-NEERI-NGP-29243/2021	EPI_ISL_1360324	B.1.36.8	1
hCoV-19/India/WB-1931500693878/2021	EPI_ISL_1419458	B.1.1.526	1
hCoV-19/India/GJ-GBRC541a/2021	EPI_ISL_1677770	B.1.36	1
hCoV-19/India/GJ-GBRC541b/2021	EPI_ISL_1677771	B.1.36	1

Table S3. The metafile of GISAID with accession number. The Supplementary data with accession number along with detailed information's can be obtained online: Supplementary Table S3.csv

Lineage	Number	Percentage	Lineage	Number	Percentage
B.1.617.2	18764	42.15	B.1.617.1	4478	10.06
AY.4	3428	7.70	B.1.1.7	3213	7.22
B.1	2640	5.93	AY.12	1016	2.28
AY.23	949	2.13	B.1.36.29	660	1.48
B.1.1	508	1.14	B.1.36	488	1.10
AY.102	440	0.99	AY.43	404	0.91
AY.16	401	0.90	AY.26	361	0.81
AY.127	343	0.77	B.1.1.306	293	0.66
B.1.1.216	278	0.62	AY.20	267	0.60
B.1.617.3	236	0.53	B.1.333	211	0.47
B.1.525	209	0.47	AY.125	208	0.47
AY.122	205	0.46	AY.61	197	0.44
AY.103	177	0.40	B.1.351	176	0.40
B.1.618	164.00	0.37	AY.120	159.00	0.36
AY.5	153.00	0.34	B	141.00	0.32
AY.44	132.00	0.30	AY.25	130.00	0.29
AY.7.1	124.00	0.28	B.1.243	122.00	0.27
B.1.1.526	118.00	0.27	AY.50	106.00	0.24
AY.106	104.00	0.23	B.1.36.8	100.00	0.22

Table S4. The percentage (%) cases of the lineages from Jan to Sept 2021 The Supplementary data with all lineages circulating in Indian population during Jan to Sept 2021 can be obtained online: Supplementary Table S4.csv

References

1. R Core Team. *R: A Language and Environment for Statistical Computing*. R Foundation for Statistical Computing, Vienna, Austria (2022). https://www.R-project.org.
2. South, A. *rnaturalearth: World Map Data from Natural Earth* (2017). R package version 0.1.0, https://CRAN.R-project.org/package=rnaturalearth.
3. Wickham, H. *ggplot2: Elegant Graphics for Data Analysis* (Springer-Verlag New York, 2016). https://ggplot2.tidyverse.org.
4. Sarkar, P. *et al.* Genome characterization, phylogenomic assessment and spatio-temporal dynamics study of highly mutated BA variants from india. *Indian J. Med. Microbiol.* DOI: 10.1016/j.ijmmb.2022.10.006 (2022).
